# Complete Genome Sequence of the First Camelpox Virus Case Diagnosed in Israel

**DOI:** 10.1128/MRA.00671-19

**Published:** 2019-08-22

**Authors:** Ofir Israeli, Inbar Cohen-Gihon, Anat Zvi, Ohad Shifman, Sharon Melamed, Nir Paran, Orly Laskar-Levy, Adi Beth-Din

**Affiliations:** a Department of Biochemistry and Molecular Genetics, Israel Institute for Biological Research, Ness Ziona, Israel; b Department of Infectious Diseases, Israel Institute for Biological Research, Ness Ziona, Israel; KU Leuven

## Abstract

We report here the whole-genome sequence of the first camelpox virus case diagnosed in Israel. The strain (Negev2016) was isolated in 2016 from a camel in southern Israel and was sequenced on the Illumina MiSeq and Oxford Nanopore MinION platforms.

## ANNOUNCEMENT

Camelpox virus (CMLV) is a double-stranded DNA virus species of the family *Poxviridae* and the genus Orthopoxvirus. It is a large, brick-shaped, enveloped virus that ranges in size from 265 to 295 nm. CMLV is the etiological agent of camelpox, a disease that causes skin lesions and a generalized infection in camels. Approximately 25% of young camels that become infected might die from the disease, while infection in older camels is generally milder (1, 2). Phylogenetic analysis shows that among orthopoxviruses (OPVs), CMLV is the closest strain to variola virus (VARV), the causative agent of smallpox (3), although each virus exhibits a strictly narrow host range. VARV exhibits exclusive human specificity, whereas CMLV infects Old World camelids, with very rare human infection cases (4, 5).

In the summer of 2016, a disease with skin manifestation was reported in camels in the southern part of Israel. Infected animals in two herds in the Be’er Sheva district were reported. Inspected herds of mainly adult females exhibited symptoms including weakness, loss of appetite, fever, abortions, and multifocal lesions on the skin. During the initial stages of the disease, stiff papules were observed, while at the advanced stage, they became ulcerated with purulent secretion. No mortalities were reported, and the inspected animals recovered after a few weeks. There were no subsequent reports of further spreading of the disease. No infections in husbandry personnel were reported. In order to identify the etiologic agent, skin samples from representative camels were subjected to various diagnostic tests (6). Transmission electron microscopy analysis revealed the presence of an OPV-like virus, based on its characteristic brick shape. The virus from the skin lesions induced a cytopathic effect in Vero cells, which were subsequently positively stained by an orthopox-specific antibody. The final identification of the virus was accomplished by two quantitative PCR (qPCR) tests (6, 7). Here, we report, for the first time, the whole-genome sequencing of a CMLV case in Israel.

For genome sequencing of the Israeli CMLV, DNA was extracted from Vero cells infected with CMLV using the QIAamp DNA blood minikit (Qiagen). A Nextera XT paired-end library (Illumina) was prepared from 1 ng of DNA. The library was sequenced on a MiSeq platform (Illumina) using paired-end sequencing reads with 150-nucleotide (nt) length and a mean insertion size of 680 nt. This produced 924,114 reads, with 13,860 coverage mapping to CMLV. FastQC was used to check the raw sequence data for quality control (https://www.bioinformatics.babraham.ac.uk/projects/fastqc/). Mapping was performed using Bowtie 2 (8), with default parameters. For Nanopore sequencing, libraries were prepared from 5 ng of genomic DNA (gDNA) using a rapid PCR barcoding kit (SQK-RPB004; Oxford Nanopore), without fragmentation, and sequenced on the MinION device using an MK1 R9.4 flow cell, following the protocol for 1D gDNA, producing 66,960 reads. A hybrid Nanopore-Illumina *de novo* assembly was performed using SPAdes (9), with the following parameters: -t 4 –m 32 –k 31,51,71. The genome ends were determined by manually adding the large inverted terminal repeats to the central genome. The complete genome of the Israeli CMLV Negev 2016 strain, which we named Negev2016, consists of 201,609 bp (G+C content, 33.2%). The genomic sequence of the CMLV Negev2016 strain was compared to all other complete CMLV genomic sequences available in the NCBI database, revealing that the sequence differs from the closest strain, CMLV Kazakhstan strain M-96, by 349 single-nucleotide polymorphisms (SNPs) (99.55% genome similarity). The CMLV sequence reported here is the first available full CMLV genome from the Mediterranean region and will enrich the existing data of CMLV sequences.

### Data availability.

The genome sequence of the Israeli CMLV isolate was submitted to NCBI GenBank and is available under the accession no. MK910851. The raw reads were submitted and are available in the Sequence Read Archive at NCBI as Fast5 files (SRA accession no. PRJNA540418).
